# ADP-Ribosylation and Antiviral Resistance in Plants

**DOI:** 10.3390/v15010241

**Published:** 2023-01-14

**Authors:** Nadezhda Spechenkova, Natalya O. Kalinina, Sergey K. Zavriev, Andrew J. Love, Michael Taliansky

**Affiliations:** 1 Shemyakin-Ovchinnikov Institute of Bioorganic Chemistry, The Russian Academy of Sciences, 117997 Moscow, Russia; 2 Belozersky Institute of Physico-Chemical Biology, Lomonosov Moscow State University, 119991 Moscow, Russia; 3 The James Hutton Institute, Invergowrie, Dundee DD2 5DA, UK

**Keywords:** PARP, PARG, PARylation, plant virus resistance

## Abstract

ADP-ribosylation (ADPRylation) is a versatile posttranslational modification in eukaryotic cells which is involved in the regulation of a wide range of key biological processes, including DNA repair, cell signalling, programmed cell death, growth and development and responses to biotic and abiotic stresses. Members of the poly(ADP-ribosyl) polymerase (PARP) family play a central role in the process of ADPRylation. Protein targets can be modified by adding either a single ADP-ribose moiety (mono(ADP-ribosyl)ation; MARylation), which is catalysed by mono(ADP-ribosyl) transferases (MARTs or PARP “monoenzymes”), or targets may be decorated with chains of multiple ADP-ribose moieties (PARylation), via the activities of PARP “polyenzymes”. Studies have revealed crosstalk between PARylation (and to a lesser extent, MARylation) processes in plants and plant–virus interactions, suggesting that these tight links may represent a novel factor regulating plant antiviral immunity. From this perspective, we go through the literature linking PARylation-associated processes with other plant regulation pathways controlling virus resistance. Once unraveled, these links may serve as the basis of innovative strategies to improve crop resistance to viruses under challenging environmental conditions which could mitigate yield losses.

## 1. Introduction to ADP-Ribose Metabolism

**Figure 1:**
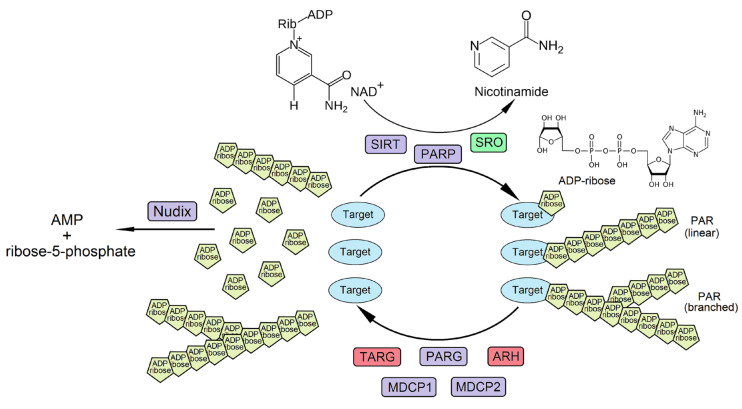
ADP-ribose metabolism mediated by poly (ADP-ribose) polymerases (PARP), silent information regulators (sirtuins, SIRT), SRO (Similar to RCD One), poly(ADP-ribose) glycohydrolases (PARG), ADP-ribosyl hydrolases (ARH), macrodomain-containing proteins (MDCPs), including MDCP1, MDCP2, terminal ADP-ribose protein glycohydrolases (TARG) and nucleoside diphosphate linked to some moiety-X (NUDIX) hydrolases (Nudix). Plant-specific proteins (SRO) are highlighted in green. Proteins (TARG, ARH) which were not found in plants are highlighted in red.

ADP-ribosylation (ADPRylation) is a well-known highly conserved reversible posttranslational protein modification which is involved in regulation of a wide range of key biological processes, such as DNA repair, cell signalling, programmed cell death, responses to biotic and abiotic stresses, and many others. Members of the poly(ADP-ribosyl) polymerase (PARP) family play a central role in the process of ADPRylation. During ADPRylation, ADP-ribose moieties are typically covalently (or non-covalently) added to target proteins, which results in either decoration of the proteins with single ADP-ribose moiety (MARylation) or with poly(ADP-ribose) (PAR) chains of different lengths and branching morphologies (PARylation) [1,2]. MARylation processes are catalysed by mono(ADP-ribose) transferases (MARTs or PARP “monoenzymes”), whereas PARylation is catalysed by PARP “polyenzymes”, both of which use nicotinamide adenine dinucleotide (NAD+) as a donor of ADP-ribose for PAR (MAR) synthesis. This reaction also leads to the formation of nicotinamide as a collateral product (Figure 1; [1,3]). The resultant ADPRylated proteins, including PARP itself and other protein targets, operate as regulators of various cellular signalling pathways. After their regulatory functions are completed, poly(ADP-ribose) glycohydrolases (PARGs) hydrolyse the PAR-chains attached to the proteins, which liberates free PAR or ADP-ribose. In mammalian cells, the process of ADPRylation can also be reversed by ADP-ribosyl hydrolase (ARH) and macrodomain-containing proteins (MDCPs), including MDCP1, MDCP2 and terminal ADP-ribose protein glycohydrolase (TARG) [1]. Subsequently, free PAR is cleaved into AMP and ribose-5-phosphate via the activity of nucleoside diphosphate linked to some moiety-X (NUDIX) hydrolases, which have specificity for ADP-ribose or ADP-ribose pyrophosphates (Figure 1; [4]).

The PARP family contains 17 mammalian proteins, of which only four are capable of generating PAR chains, with PARP1 being the best studied and most abundant member of this group [5]. Numerous reports have placed PARP1 and related PARP proteins at the interface between various stress signalling pathways in mammalian cells, including genotoxic, inflammatory, oxidative and metabolic stress responses [5]. PARP1 is a nuclear enzyme, and its activities as well as PAR-mediated events, are intimately tied to nuclear compartments. Of note are interactions of PARP1 and PARylated proteins with the nucleolus and Cajal bodies (CBs). The nucleolus is the prominent subnuclear compartment in the cell nucleus, which is traditionally recognised as a site of ribosomal RNA synthesis and ribosome biogenesis. CBs are another type of subnuclear structure which are functionally and physically connected to the nucleolus. CBs have been implicated in various RNA-related processes, such as biogenesis, maturation, and recycling of small nuclear (snRNA), small nucleolar (snoRNA) and small Cajal body (scaRNA) RNAs, histone mRNA processing and telomere maintenance [6]. In addition, there is a growing body of evidence that CBs are also involved in cellular functions including growth and development, telomerase activity, gene silencing and responses to pathogen attacks and abiotic stresses [7]. Unmodified PARP1 molecules are normally localised in the nucleolus and bind to chromatin. However, PARP1 automodified by PARylation and other additional PARylated proteins have been shown to interact non-covalently via PAR polymers with coilin, the major scaffolding protein of CBs [8]. This association likely mediates trafficking of the PARP1 and other PARylated target proteins from chromatin and the nucleolus into CBs for PAR removal and recycling by PARG. Given that CBs play an important role in various stress responses it seems conceivable that the interaction of PARP1/PARylated proteins with coilin/CBs may provide a mechanism which regulates PARylation levels in order to activate various responses to pathogen attacks, abiotic stresses and developmental cues [6,7].

Interestingly, despite its potentially misleading name, most other PARP proteins possess MARylation activity. In mammalian cells, MARylation may play a role in immunity, transcription, regulation of the cytoskeleton and various stress responses including those occurring in the endoplasmic reticulum, which have recently been reviewed in detail by Challa et al. [2].

With regard to the substrate specificity of ADPRylation, it is worth noting that protein targets can be modified via PARylation or MARylation at a wide variety of amino acid residues such as Ser, Asp, Glu, and Cys, constituting a diverse repertoire of ADPRylation targets [2,9,10].

In addition to proteins as a substrate for ADPRylation, it has recently become evident that some PARPs are capable of modifying nucleic acids. Mammalian PARPs can transfer ADP-ribose onto the 5′- or 3′-ends of both DNA and RNA nucleic acid substrates, if they possess either a 5′- or a 3′-phosphate [2,10,11]. Several bacterial ADP-ribosyltransferases such as scabins have been demonstrated to modify DNA internally [10,12]. Functional roles for nucleic acid ADP-ribosylation are largely uncharacterised but some properties may be extrapolated from findings observed on other organisms. Pierisin from cabbage butterfly species, *Pieris rapae* and pierisin-like proteins and scabins from Streptomyces bacteria act as cellular toxins and possess ADPRylation activity that mainly target DNA. Such bacterial DNA-modifying ADP-ribosyltransferases (toxins) are typically counteracted by ADP-ribosyl hydrolases (antitoxins or immunoproteins) residing in the same operon [10,12]. This suggests that the ADPRylation system in pathogenic bacteria could influence their interactions with different hosts to modulate defence responses (and toxic effects), while the bacteria themselves are protected against these toxic activities. Similar toxin (ADP-ribosetransferase)-antitoxin(ADP-ribosehydrolase) interactions affecting host defence responses occur in the case of bacterial protein-targeting ADP-ribosetransferases, such as diphtheria/diphtheria-like or cholera toxins [12].

ADPRylation events (both MARylation and PARylation) are also known to be a significant factor involved in the development of responses to highly diverse group of RNA- and DNA-containing human and animal viruses, including Epstein–Barr virus (EBV), human immunodeficiency virus 1, the herpesviruses, human cytomegalovirus, hepatitis B virus, Ebola virus, Marburg virus, coxsackievirus B3, Japanese encephalitis virus, Sindbis virus, influenza A virus, poliovirus, dengue virus, Zika virus, herpes simplex virus 1, vesicular stomatitis virus, chikungunya virus and SARS-CoV-2 [13,14,15]. Interestingly, ADPRylation may differentially modulate virus infections by either enhancing [14,15] or inhibiting [13] virus propagation. Host defence responses against viruses, may be achieved either by direct antiviral activity, such as degradation of viral RNAs, targeting of viral proteins, inhibition of replication or translation, or by indirect antiviral activity via activation of the innate immune response [13]. Examples of direct antiviral activities include exosome-mediated degradation of target viral RNAs by PARP13 (devoid of catalytic activity) and PARP7 (MARylation); inhibition of viral genome replication (RNA transcription) by PARP12 (MARylation); regulation of virus transcription by PARP1 (PARylation), PARP2 (PARYylation) and PARP5 (PARylation), which may PARylate or directly interact with the EBV EBNA1 protein, preventing its binding to the OriP promoter, and thus inhibiting EBV replication; suppression of viral translation by PARP7, PARP12 and PARP13, which can block the formation of the translation initiation complex on viral mRNA; and MARylation of viral proteins by PARP12, leading to their proteosomal degradation [13]. The role of PARPs as positive regulators of immune responses has been demonstrated for PARP7, PARP9 (MARylation), PARP10 (MARylation), PARP11 (MARylation), PARP12, PARP13 and PARP14 (MARylation), and has been discussed in detail in [13].

Another interesting link between ADPRylation and antiviral immunity may come from findings demonstrating that both DNAs and RNAs can be MARylated by various PARPs. It is, therefore, tempting to suggest that such a process could provide a very efficient way to suppress viral replication via the targeting of viral RNA or DNA. In this connection, the macrodomains present in certain viruses could operate as antagonists to prevent ADP-ribosylation of viral nucleic acids, thus representing a counterdefence strategy that facilitates virus replication. Similarly, as a reaction to coronaviral infection, the activity of human antiviral PARPs (PARP9, PARP12 and PARP14) is significantly upregulated and induces the proinflammatory defence response in the cell. Virus-encoded macrodomains can reverse this antiviral defence and enhance virus infection [12]. With regards to other pro-viral activities, it has been shown that PARP1/PARylation is required for replication by certain viruses, such as human cytomegalovirus or influenza A virus [14,15]. The mechanism underlying such pro-viral activity includes interaction between PARylated proteins including automodified PARP, and some viral components, leading to suppression of host type I interferon responses to viral infections. Curiously, the bacterial CRISPR-Cas immune system could also be inactivated by a newly identified ADP-ribosyltransferase from bacteriophage AcrIF11 [12].

Collectively, these observations clearly show that the process of ADPRylation plays a critical role in mediating responses to biotic (virus attack) and possibly abiotic stresses.

## 2. ADP-Ribosylation in Plants

### 2.1. Major Enzymes

In contrast to mammals, the Arabidopsis genome contains only three genes encoding PARP proteins, PARP1, PARP2 and PARP3. Two of them, PARP1 and PARP2, are nuclear and have been demonstrated to be bona fide poly(ADP-ribose) polymerases that mediate PARylation reactions [1,16,17]. The functional role of plant PARP3 remains largely unknown. It is worth noting that until recently, the nomenclature of plant PARP1 and PARP2 genes was inconsistent. However, according to the phylogenetic relationships and structural similarities with animal counterparts, the classification of Arabidopsis PARP proteins has now been standardized and PARP1, PARP2 and PARP3 genes have been allocated to At2g31320 (formerly designated as PARP2), At4g02390 (formerly designated as PARP1) and At5g22470 gene accessions, respectively. Based on the domain architecture of these proteins, Arabidopsis PARP1 and PARP2 resemble human HsPARP1 and HsPARP2, respectively, whereas PARP3 has no counterpart in humans [1,16]. Sirtuins (silent information regulators, SIRTs) are another group of mono-ADP-ribosyltransferases (which in addition, possess NADH-dependent deacetylase activity) (Figure 1). They are present in bacteria, plants and animals. In plants, sirtuins may be involved in mitochondrial energy metabolism, signalling pathways governed by plant hormones including auxin, and plant immunity [18]. In addition to the two described groups of ADP-ribose transferase groups mentioned above, higher plants have a plant-specific family of proteins containing non-canonical PARP-like domains, called the SRO (Similar to RCD One) proteins which refer to the RCD1 (Radical-induced Cell Death 1) protein of *Arabidopsis* (Figure 1). However, there are some contradictory data on whether or not they have ADPRylation activity and uncertainty as to how this relates to biological function, particularly when different SRO members are considered. For example, SRO2 has recently been shown to possess MARylation activity, which ensures appropriate immune and stress responses [19], in contrast with SRO1 for which it is unknown whether its capacity to confer salinity tolerance in wheat is contingent on its possible ADPRylation activity [20,21].

In contrast to the mammalian genome which contains only one PARG gene, Arabidopsis has two PARG genes (At2g31870 for PARG1 and At2g31865 for PARG2). The products of both these genes possess canonical PARG catalytic activity in vivo and in vitro (Figure 1; [16]). In addition, some Arabidopsis proteins show considerable sequence homology to the human macrodomain-containing proteins (MDCP1 and MDCP2) mentioned above, and consequently *in planta* they may fulfil analogous functions (Figure 1; [1]). Similar to their mammalian counterparts, plant genomes contain a diverse superfamily of NUDIX hydrolases, six of which (NUDIX2, 6, 7, 10, 14, and 19) in Arabidopsis have the enzymatic pyrophosphohydrolase activities toward ADP-ribose [22], and can therefore also be considered as important components in the whole PARylation process. Interestingly, in Arabidopsis there seems to be an absence of homologs to the human ARH and TARG proteins.

### 2.2. Biological Functions

**Figure 2:**
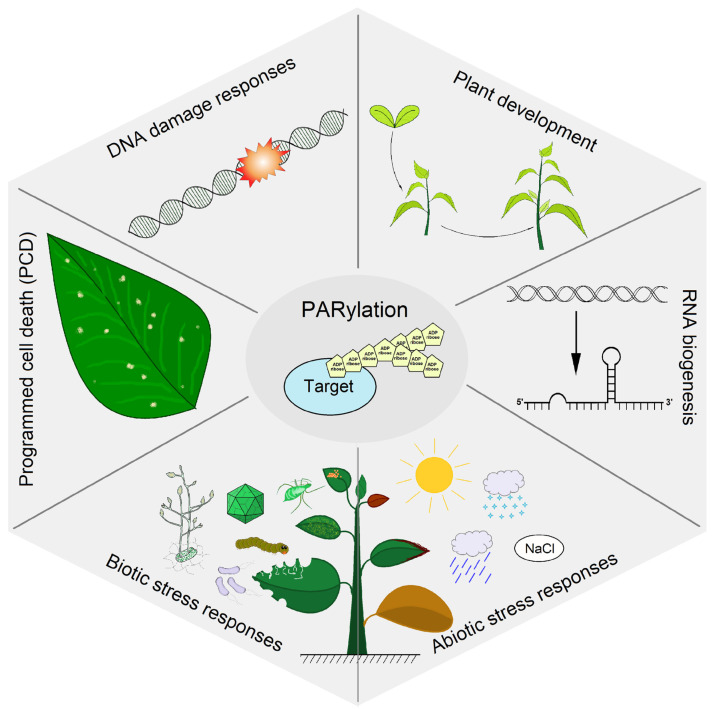
The role of the PARylation in regulating plant functions.

Plants, like animals, regulate multiple cellular functions via the PARylation process (Figure 2):

#### 2.2.1. DNA Damage Responses

One of the best-studied functions of PARylation is DNA repair. The expression of the PARP proteins and their subsequent activation are both induced by treatment with DNA-damaging agents, such as ionizing radiation, zeocin (a radiomimetic drug that induces breaks in DNA) or cisplatin (an inhibitor of DNA replication) in Arabidopsis [1]. In agreement with increased gene expression and activity, the genetic inhibition of PARP genes essentially enhanced the sensitivity of plant growth and development to DNA damaging agents such as methyl methane sulfonate, bleomycin and mitomycin [1]. In addition to these abiotic DNA degrading factors, DNA damage caused by the bacterial pathogen Pseudomonas syringae pv. Tomato (Pst) was also enhanced in parp1 and parp2 mutant plant lines [1]. Collectively these observations indicate that PARP deficiency may increase susceptibility to DNA damage, suggesting that the PARylation process is involved in plant genotoxic stress responses.

At lower levels of DNA damage, PARP proteins recruit some DNA damage factors via PARylation processes to the DNA [1,16,23]. Such damage factors include classical and alternative NHEJ (non-homologous end joining), BER (base excision repair), HR (homologous recombination), DNAP (DNA polymerase), DNA-PKcs (DNA-dependent protein kinase) and many others [23]. It seems that PAR chains may act as docking platforms for the recruitment of such DNA repair complexes. Interestingly, the termini of DNA strand breaks can also be PARylated by PARP proteins [24], suggesting that PARylated ends of recessed DNA duplexes may operate in concert with PARylated DNA damage protein factors to enforce the process of DNA repair. With very severe DNA damage, PARP overexpression mediates various forms of programmed cell death (PCD), such as apoptosis or necrosis, which are typically activated by excessive formation of PAR [16].

#### 2.2.2. Plant Development

In addition to its role in DNA repair, PARP proteins and PARylation are involved in regulation of plant development [1]. For instance, the formation of tracheary elements in pea roots and artichoke tissue culture has been reported to be suppressed by the PARP inhibitor, 3-aminobenzamide (3AB) [1]. In addition, Arabidopsis seed germination was reduced and delayed in PARP1 and PARP2 knockdown lines. On the other hand, in some other cases, PARP-deficient Arabidopsis mutants did not demonstrate any apparent developmental changes compared with wild type plants [1]. These observations indicate that effect of PARylation on plant development may be highly dependent on conditions and further research is required to solve currently existing discrepancies.

#### 2.2.3. RNA Biogenesis

Apart from DNA repair, PCD, and development, an emerging body of evidence implicates PARP and PARylation in many different stages of RNA biogenesis, metabolism and protein biosynthesis such as transcriptional initiation, polymerase II elongation and termination, chromatin remodelling and decondensation, co-transcriptional alternative splicing, RNA polyadenylation and mRNA export, RNA stabilization, ribosome biogenesis and translation [11,25]. Mechanisms underlying these activities include multiple discrete interactions of PARP with proteins, DNA, RNA as well as PARylation of target molecules and autoPARylation. Moreover, recent studies demonstrate that RNA can be directly ADP-ribosylated by PARP proteins. It is quite conceivable that dysregulation of PARP activities in these processes can cause plant diseases.

Another intriguing finding is that the PARP proteins may be involved in the assembly and maintenance of some subnuclear compartments such as the nucleolus and Cajal bodies, which are responsible for processing, assembly and transport of ribonucleoprotein (RNP) complexes [8,25,26] which may provide additional regulatory mechanisms which control RNA-dependent processes.

#### 2.2.4. Abiotic Stress Responses

Plants are constantly exposed to different types of environmental (abiotic) stresses that can adversely affect their growth and development. These negative influences are likely to be further aggravated as a consequence of future global climatic changes, which will increase the risk of environmental stresses. Plants perceive abiotic stress signals and produce an appropriate response with altered metabolism, growth and development. At the molecular level, abiotic stress tolerance can be achieved by altering the accumulation of osmoprotectants, production of chaperones, superoxide radical scavenging mechanisms, exclusion or compartmentalisation of ions by efficient transporter and symporter systems [27]. Over the past decade, various approaches have been employed to understand the basis of stress tolerance and identify the genes that are involved in the stress response.

Silencing of PARP genes in Arabidopsis and oilseed rape using RNA interference increased the tolerance to drought, high light, heat and methyl viologen (paraquat) treatment, accompanied with a decreased PARP activity and PAR accumulation [1,4]. The mechanism of such tolerance is largely uncharacterised. One possibility is that the excessive energy consumption, which is a normally associated with PARP activation under stress conditions was prevented in the PARP silenced lines [28]. Another possibility is based on findings that PARP silencing results in increase in amounts of abscisic acid (ABA) and upregulation of ABA-responsive genes [28]. ABA is the one of the major plant hormones which is involved in plant abiotic stress responses [29]. These data suggest that PARP proteins or PARylation could be implicated in abiotic stress responses as a negative transcriptional regulator in an ABA-dependent way. In agreement with these data, analysis of Arabidopsis parg knockout mutant exhibited high sensitivity to drought, oxidative and osmotic stress, suggesting that PARG and reduced PARylation rates are required for tolerance to abiotic stresses [16].

#### 2.2.5. Plant Immunity

Pathogen-associated molecular pattern (PAMP; pathogen molecules recognised by specific plant receptors) triggered immunity (PTI) constitutes the first layer of plant immunity that restricts pathogen invasion [30]. The N-terminal 22 amino acids of flagellin (flg22) from Pseudomonas bacteria and the N-terminal 18 amino acids of EF-Tu (elongation factor Tu, elf18) from Escherichia coli are typical bacterial PAMPs. They mediate immune responses including the oxidative burst, elevation of cytosolic free calcium, callose deposition on cell walls, activation of mitogen-activated protein kinases (MAPK) and transcriptional activation of defence genes, such as genes encoding pathogenesis related (PR) proteins [31]. PTI is also tightly related to phytohormones (in particular salicylic acid, SA)-mediated signalling. Another important factor controlling PTI in plants is lysine 63 (K63) linked ubiquitination, which does not usually lead to the 26S proteasome-dependent protein degradation as in the case of K48 associated ubiquitination, but instead it is involved in the coordination of various cellular processes such as macromolecular trafficking, protein folding and maturation and signaling [32].

Pharmacological studies using the PARP inhibitor 3AB have been shown to block a distinct subset of late responses characteristic of the flg22 and elf18 linked PTI such as callose deposition, whereas early responses such as the burst of reactive oxygen species (ROS) and early responsive gene expression are not affected [33,34]. Transcriptomic analyses further revealed transcriptional responses in Arabidopsis that were altered by 3AB [35].

In contrast to pharmacological studies, genetic knockout experiments showed that Arabidopsis parp mutants exhibit a variety of different responses to flg22 or elf18 treatments including reduced, enhanced or unaffected callose deposition depending on plant age or experimental conditions [1]. These contrasting results could indicate that the different parp mutants have complex and divergent impacts on plant phenotypes under the test conditions, and also that the PARP inhibitors may have potential off-target effects (see below).

The more convincing evidence showing direct interplay between PARylation and plant immunity (via ubiquitination) was recently published by Yao et al. [32]. It was shown that Arabidopsis PARPs/PARGs physically interact with ubiquitin-conjugating enzymes (UBC13), the main players of K63-associated polyubiquitination, and protein disulfide-isomerases (PDIs) that ensure proper folding and maturation of proteins destined for secretion including PR proteins. More remarkably, these interactions were significantly facilitated by flg22 and led to enhanced PARylation of UBC13 and PDI which in turn plays critical role in proper folding and secretion of PR proteins and immune signalling. Another report linking plant immunity, ADPRylation and ubiquitination [19] demonstrated that immune elicitation with Pseudomonas syringae pathogen effector HopU1 promotes MARylation of Arabidopsis immune regulators SZF1/SZF2 by the non-canonical ADP-ribosyltransferase SRO2. MARylation antagonizes polyubiquitination of SZF1 and thereby stabilized the SZF1 proteins. These observations suggest coordinated interplay between plant ADPRylation, ubiquitination, protein folding and secretion, and plant immunity.

It is also worth noting that some plant bacterial pathogens such *as Streptomyces scabies* encode scabins, a mono-ADP-ribosyltransferase toxins which target DNA molecules [36]. However, whether scabin-mediated ADPRylation of plant DNA molecules plays a role in plant-pathogen interactions remains to be elucidated. On a more general level, it is of great interest to explore if plant pathogen ADP-ribosyltransferases (like scabins) can ADPRylate nucleic acids (DNA/RNA) and if so, what is their function in modulating plant immunity?

#### 2.2.6. Possible Off-Target Effects of PARP Inhibitors

Experimental data obtained employing pharmacological PARP inhibition often contradict with genetic (knockout) results [1,37]. It is particularly obvious with monitoring plant cell wall enforcement with callose induced by PTI elicitors. As mentioned above, 3AB consistently inhibits callose deposition elicited by flg22 and elf18, although it is not the case with the parp1parp2parp3 mutant line [37]. Moreover, other PARP inhibitors such as PJ-34 and INH2BP inhibit PARP activity in Arabidopsis but do not block PAMP-induced callose deposition. These data point to potential nonspecific off-target action of at least some PARP inhibitors in plants. This suggestion is supported by a transcriptomic analysis in which a treatment with two different PARP inhibitors, 3AB and 3-methoxybenzamide (3MB), modulated the expression of 228 and 3935 genes, respectively [35]. Such a great difference makes it highly unlikely that the effects are caused by the inhibition of canonical PARP proteins alone. Collectively, these observations show that results on the role of PARylation in processes in plants must be interpreted with great caution.

## 3. PARylation and Plant–Virus Interactions: What Is Already Known?

While much remains to be discovered, several lines of compelling evidence point toward the suggestion that PARylation plays important role in plant virus interactions. The first indication of the functional links between PARylation and plant virus infections came from the observation that PARG2 transcription was significantly upregulated in cucumber mosaic virus (CMV) infected Arabidopsis plants carrying an RCY1 gene which confers extreme resistance to CMV [38].

In our work, we observed a consistent and concerted upregulation of various PAR-degrading enzymes including PARG, NUDIX and ADP-ribose pyrophosphatases (ADP-PPase) in response to infection of potato plants with potato virus Y (PVY). According to these changes, PAR accumulation was significantly increased upon PVY infection compared to non-infected plants. It is thus conceivable to suggest that the PVY–plant interactions are at least partially regulated by PARylation functions [39].

In more recent work we showed that an exogeneous PVY-specific fragment of double stranded RNA (dsRNA) protects potato against PVY [40]. Usually, the antiviral effect of exogenous dsRNA is attributed to activation of RNA-silencing mechanisms. However, it has also been suggested that dsRNA may be involved as a virus-specific elicitor in PTI induction [41]. PTI is nonspecific process directed against a certain virus, and therefore we tested the impact of exogenously applied PVY-derived dsRNA on infection with both a homologous virus (PVY) and an unrelated virus (potato virus X, PVX). It was found that some PTI-related genes such as WRKY29 (WRKY transcription factor 29; molecular marker of PTI), RbohD (respiratory burst oxidase homolog D), EDS5 (enhanced disease susceptibility 5), SERK3 (somatic embryogenesis receptor kinase 3)-encoding brassinosteroid-insensitive 1-associated receptor kinase 1 (BAK1), and PR-1b (pathogenesis related gene 1b) were significantly upregulated independently of the type of virus used for infection (PVX or PVY) [40]. More significantly, in addition to PTI-inducing activities, we also showed that PVY-specific dsRNA is able to upregulate production of PARG, which therefore could be regarded as a positive regulator of antiviral responses. Thus, it seems that exogenous dsRNA application is a multi-faceted technology which mainly triggers RNA silencing mechanism, the major virus resistance pathway, and also induces PTI/PAR-based mechanisms which may represent a safeguard strategy in case the RNA silencing fails for some reason(s).

In many cases, the nucleolus and CBs play a crucial role in the infection cycle of DNA and RNA-containing viruses [6,7,42,43,44,45,46]. Various plant virus proteins interact with the nucleolus and CBs (modifying their structure and architecture), and their major protein components, such as fibrillarin or coilin, respectively, to recruit them for various invasive processes such as virus movement or silencing suppression [6,7,46]. At the same time, plants may exploit these interactions to develop antiviral defence responses [44,45], suggesting that subnuclear compartments may differentially modulate plant responses to virus infections. As noted above, the nucleolus and CBs are also tightly associated with the PARylation process. PARP1 directly interacts with both coilin and fibrillarin, and such interactions are required for trafficking of PARylated proteins from the nucleolus to CBs for their recycling [8]. Moreover, PARP proteins may be involved in modulating the formation, maintenance and content of the nucleolus and CBs [26], which could influence their interactions with viruses.

Thus, the data presented above strongly suggest that PARylation processes may be linked to how animal and plant hosts respond to virus infection, although such interplay in plants is less well studied.

## 4. PARylation and Plant–Virus Interactions: What Is Next?

Viruses represent one of the major plant pathogens (biotic stress factors), causing almost half (47%) of all emerging plant disease outbreaks (more than any other pathogen group; [47]). With the world’s population expected to reach more than 9 billion by 2050, new innovative strategies and technologies which protect plants against viruses are required to help us face global food security issues and climate change risks. Viruses are intracellular pathogens with small genomes, which rely on host cell functions and cellular machinery to aid their own replication. Elucidating the interaction between host cell and virus components is critical for understanding the mechanisms of virus infection and for the development of novel strategies to control virus diseases. Breakthroughs in research of PARylation, as a new factor controlling plant–virus interactions, may allow us to develop such approaches/technologies to mitigate adverse impacts of viruses on crops. This is a continuous pursuit that should follow some guidelines:

- CBs and their signature protein, coilin, have been shown to affect a variety of interactions between host plants and viruses that have RNA or DNA genomes. Moreover, the effects of coilin on these interactions are manifested differently: coilin contributes to plant defence against some viruses, but in contrast may serve to increase virus pathogenicity in other viruses. These findings show that interactions with coilin (or CBs) may involve diverse mechanisms with different viruses, and that these mechanisms act at different phases of virus infection [44,45,46]. Taking into account that CBs/coilin may trigger responses to virus infection via interaction with PARP and modulation of its activity, it would be conceivable to select a wide range of DNA and RNA viruses to address their ability to multiply in parp, parg or nudix knockout or knockdown plants.
- In nature, plants are usually exposed to diverse environmental stresses, which may modulate plant–virus interactions [48]. It is suggested that responses to abiotic stresses and virus infections may be integrated in a specific consolidated network which controls plant sensitivity to multiple stresses. Given that PARylation process may differentially affect various biotic and abiotic stresses, investigation of effects of combined stress caused by viruses and major environmental cues on parp, parg or nudix knockout or knockdown plants must be carried out. In addition to using genetic approaches, PARP inhibitors may also be exploited to explore interactions of these different molecular switches, but with the precautions discussed above.
- To elucidate the mechanisms underlying the cross-talk between PARylation and virus infection outcomes, analysis looking at expression level (transcript levels and protein levels) and activity of PARylation-related enzymes (PARP, SRO, PARG etc) and PAR itself should be performed during the infection process. It would also be important to look at possible relocalisation of PARylation related proteins potentially induced by various viruses, as significantly altered distribution (including perhaps sequestration to defined cellular regions) may greatly affect PARylation efficiency. It is well known, for example, that the nucleolus can act as a sequestration centre which may capture and detain various cellular factors [49].
- In mammalian model systems, it appears that MARylation is required for host cells to respond to viral infection. It is, therefore, conceivable to examine the distinctive role of MARylation (compared with PARylation) in plant–virus interactions.
- It has recently been shown that nucleic acids (DNA and RNA) may be directly ADPRylated. Therefore, future studies should examine whether such modifications can occur with plant viral RNAs or DNAs and investigate the biological relevance of such process.
- It would also be critically important to identify PARylation targets among virus components (proteins, nucleic acids) and host signalling molecules involved in plant responses to virus attack or to combined stresses. Application of systems biology approaches to analyse the data obtained will allow the identification of target genes and regulatory hubs which could be exploited to develop improved plant resistance to biotic and environmental stresses.
- On a practical level, genetic alteration of the PARylation (and possibly MARylation) process (by affecting PARP and PARG proteins) in crops may be achieved either by CRISPR-Cas technology (gene knock out or knock in or by alteration of a specific gene sequence) [50] or by RNA interference (RNAi)-based methods [51]. It is worth noting that for modification of genes critical to plant survival, CRISPR-Cas may not be ideal, as it would permanently affect those genes, possibly interfering with plant growth and development. Alternatively, spray induced gene silencing (SIGS) RNAi techniques which employs external treatment of plants with dsRNA as a trigger for RNAi, may be preferable for those genes since it would allow flexible reversible modification of gene expression in real time without compromising their essential function over the lifecycle of the plant. In animals MARylation often mediates host defence responses. Inhibitors of hydrolases (such as PARG) are therefore being pursued as a potential antiviral therapeutic strategy. A similar strategy could be applied for crop protection. Despite the certain off-target effects of known PARP/PARG inhibitors, development of the next generation inhibitors is required to enhance plant stress tolerance resulting in improved growth and yield.

## Data Availability

Not applicable.
